# Iron deficiency or anemia of inflammation? Differential diagnosis and mechanisms of anemia of inflammation

**DOI:** 10.1007/s10354-016-0505-7

**Published:** 2016-08-24

**Authors:** Manfred Nairz, Igor Theurl, Dominik Wolf, Günter Weiss

**Affiliations:** 1 Department of Internal Medicine VI, Infectious Diseases, Immunology, Rheumatology, Pneumology, Medical University of Innsbruck, Anichstraße 35, 6020 Innsbruck, Austria; 2 Medical Clinic III, Department of Oncology, Hematology and Rheumatology, University Clinic Bonn (UKB), Bonn, Germany

**Keywords:** Anemia of inflammation, Anemia of chronic disease, Iron, Hepcidin, Macrophage, Entzündungsanämie, Anämie bei chronischer Erkrankung, Eisen, Hepcidin, Makrophage

## Abstract

Iron deficiency and immune activation are the two most frequent causes of anemia, both of which are based on disturbances of iron homeostasis. Iron deficiency anemia results from a reduction of the body’s iron content due to blood loss, inadequate dietary iron intake, its malabsorption, or increased iron demand. Immune activation drives a diversion of iron fluxes from the erythropoietic bone marrow, where hemoglobinization takes place, to storage sites, particularly the mononuclear phagocytes system in liver and spleen. This results in iron-limited erythropoiesis and anemia. This review summarizes current diagnostic and pathophysiological concepts of iron deficiency anemia and anemia of inflammation, as well as combined conditions, and provides a brief outlook on novel therapeutic options.

## Zusammenfassung

Eisenmangel und Immunaktivierung sind die zwei häufigsten Ursachen der Anämie. In beiden Situationen besteht ursächlich eine Störung der Eisenhomöostase. Die Eisenmangelanämie beruht auf einer Verminderung des Gesamtkörpereisens infolge von Blutverlust, unzureichender alimentärer Zufuhr oder intestinaler Absorption bzw. erhöhtem Bedarf an Eisen. Immunaktivierung führt bei normalem Gesamtkörpereisen zu dessen Umverteilung vom erythropoetischen Knochenmark, der primären Stelle der Hämoglobinproduktion, in das mononukleäre Phagozytensystem der Leber und Milz, die Hauptorgane der Eisenspeicherung. Dies führt letztlich zur Anämie. In dem vorliegenden Übersichtsartikel werden aktuelle diagnostische und pathophysiologische Konzepte von Eisenmangelanämie, Entzündungsanämie sowie kombinierter Anämie zusammengefasst und ein kurzer Ausblick auf neue Therapieoptionen geboten.

## Introduction

Iron deficiency (ID) can occur in two major forms: absolute and functional ID. Both forms of ID can manifest either isolated or combined, and will result in iron-deficient erythropoiesis and, if unrecognized or left untreated, in anemia [1, 2].

**Fig. 1:**
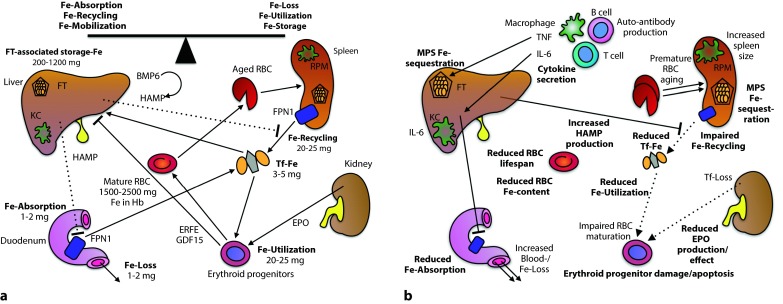
**a** Under homeostatic conditions, the absorption of 1–2 mg of iron per day compensates for its loss via desquamation of epithelial cells from skin and mucosal membranes and during menstrual bleeding. The majority of the 20–25 mg of iron required for daily erythropoiesis is provided by the degradation of effete RBC and the iron contained within their Hb (Hemoglobin). Both duodenal iron absorption and iron recycling in spleen and liver are negatively regulated by HAMP (Hepcidin antimicrobial peptide). HAMP is mainly generated by hepatocytes in response to an increase in serum iron or storage iron while erythropoietic activity inhibits HAMP expression via soluble mediators including GDF15 and ERFE. **b** Following immune activation by pathogen- or damage-associated molecular patterns, the interaction of myeloid cells with T and B lymphocytes results in the generation of pro- and anti-inflammatory cytokines. These divert iron fluxes from the circulation to storage sites by controlling the expression of HAMP, of iron transporters, and of the iron-storage protein FT. Therefore, duodenal iron absorption and macrophage iron recycling are reduced, the serum becomes iron-starved and the erythron lacks sufficient iron for proliferation and hemoglobin synthesis. Therefore, zinc may replace iron as the central heme-cation. Zinc protoporphyrin-IX (not depicted) can be measured to confirm the presence of this mechanism of iron sequestration. In addition to cytokines, other mediators such as auto-antibodies and reactive intermediates can tag mature RBC for degradation or damage them or their precursors, contributing to the hyporegenerative nature of AI. In parallel, renal EPO production is reduced and the responsiveness of the erythron to EPO is dampened. In the end, a mild to moderate normocytic anemia with evidence of iron-restricted erythropoiesis (low TSAT, high FT, low reticulocytes, high ZnPP-IX in reticulocytes, low to normal EPO) occurs. Key pathways for the pathogenesis are in boldface. Putative additional pathways are in lightface. *BMP6* bone morphogenetic protein-6, *ERFE* erythroferrone, *EPO* erythropoietin, *FPN1* ferroportin-1, *FT* ferritin, *GDF15* growth differentiation factor-15, *HAMP* hepcidin anti-microbial peptide, *IL* interleukin, *KC* Kupffer cell, *MPS* mononuclear phagocyte system, *PDGF-BB* platelet-derived growth factor isoform BB, *RBC* red blood cell, *RPM* red pulp macrophage, *Tf-Fe* transferrin-bound iron, *TNF* tumor necrosis factor, *ZnPP-IX* zinc protoporphyrin-IX

Absolute ID, as defined by a decrease in the body’s iron content, usually develops when the absorption of dietary iron in the duodenum and proximal jejunum (Fig. 1a) cannot compensate for an increased iron demand or blood loss. Despite adaptive induction of expression of the transmembrane iron transporters divalent metal transporter (DMT)-1 and ferroportin (FPN)-1 in enterocytes upon ID, iron absorption can only be increased by 2‑ to 3‑fold to approximately 5 mg per day [3, 4]. Due to this relatively inefficient process, iron stores, particularly ferritin-associated iron in liver and spleen, can become depleted during chronic bleeding episodes, repetitive blood donations, helminth infestations, through materno-fetal transfer, or during growth [5].

In very rare cases, genetic mutations of iron homeostasis proteins such as DMT1 or TMPRSS6 (Transmembrane Protease, Serine 6), the latter encoding for matriptase-2, can result in inadequate iron absorption and development of anemia [6, 7]. Similarly, lack of the iron-carrying serum protein transferrin (TF), due to genetic deficiency, auto-antibody production, or proteinuria, can cause absolute ID [8]. Inadequate iron absorption has also been found in association with *Helicobacter pylori* infection, hypergastrinemia, celiac disease, or vitamin D deficiency [9, 10]. Prolonged ID results in the inability to regenerate skin and mucosal membranes and in iron deficiency anemia (IDA) with its classical symptoms such as fatigue. Details on the clinical implications of ID are reviewed elsewhere in this special issue.

Functional ID has a more complex pathophysiology and is commonly defined as a redistribution of iron from the key sites of its utilization (erythron, epidermis, mucosal surfaces) to storage sites, particularly the hepatic and splenic mononuclear phagocyte system (MPS). Moreover, in states of increased erythropoiesis such as during therapy with erythropoiesis-stimulating agent (ESA) or after major blood loss, erythropoiesis may become iron-restricted so long as the mobilization of storage iron cannot catch up with its demand for hemoglobin (Hb) synthesis (see the interpretation of CHr (Content of reticulocyte hemoglobin), HYPO (Hypochromic erythrocytes), and ZnPP (Zinc protoporphyrin) in diagnostic section). The ultimate consequence of these functional disturbances of iron homeostasis is anemia, which is often referred to as anemia of inflammation (AI) or anemia of chronic disease (ACD).

Absolute and functional iron deficiency may also coexist. Such combined conditions render the interpretation of erythrocyte indices and parameters of iron status challenging. While new diagnostic parameters are not yet readily used in clinical routine, this differential is important as the therapeutic approach varies. In addition, the random detection of AI during routine blood sampling should prompt a search for the underlying disease.

## Iron deficiency anemia

**Fig. 2:**
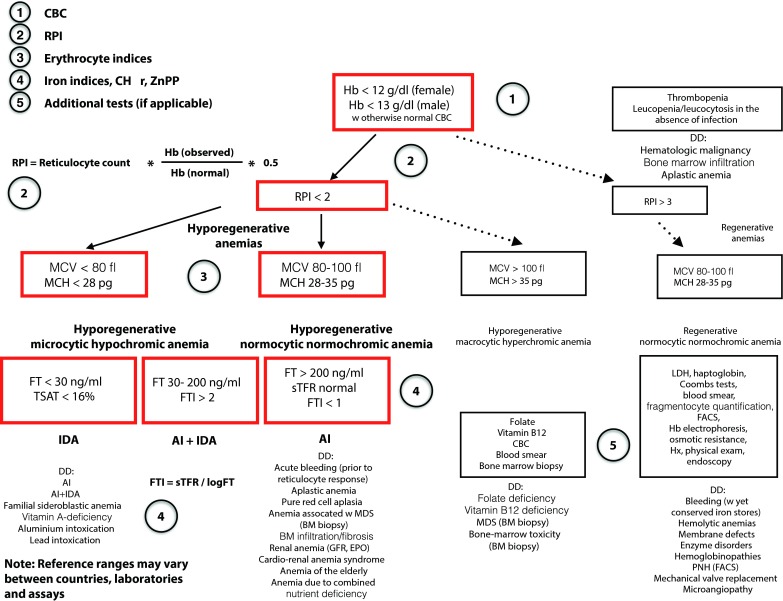
For the differential diagnosis of IDA vs. AI vs. a combination of both forms or other causes of anemia, a stepwise approach is proposed. A CBC enables the differentiation of isolated anemias from bi- and pancytopenias [161]. The latter may require a more extensive work-up. Also, the RPI can be estimated from the CBC. An RPI of <2 characterizes hyporegenerative anemias while am RPI > 3 is observed in regenerative forms such as the hemolytic anemias. Two out of three erythrocyte indices are relevant, i. e., the MCV and the MCH, as they allow for the classification of microcytic hypochromic, normocytic normochromic, and makrocytic hyperchromic anemias. In IDA, both serum FT and TSAT are reduced. In contrast, an increased FT is typical of AI. In combined conditions, the FTI, as calculated from the serum TFR divided by the logarithmic serum FT, continues to be helpful for the differential diagnosis. In the future, novel parameters such as HAMP may be incorporated into diagnostic algorithms. Note: Reference ranges may vary between countries, laboratories, and assays. Hb cutoffs correspond to WHO definitions. *AI* anemia of inflammation, *BM* bone marrow, *CBC* complete blood count, *DD* differential diagnosis, *EPO* erythropoietin, *GFR* glomerular filtration rate, *FACS* fluorescence activated cell sorting, *FT* ferritin, *FTI* ferritin index, *Hb* hemoglobin, *Hx* history, *IDA* iron-deficiency anemia, *LDH* lactate dehydrogenase, *MCH* mean corpuscular hemoglobin, *MCV* mean corpuscular volume, *MDS* myelodysplastic syndrome, *PNH* paroxysmal nocturnal hemoglobinuria, *RPI* reticulocyte production index, *sTFR* soluble transferrin receptor, *TSAT* transferrin saturation

While IDA poses a major public-health problem in developing countries [11], it is also frequently observed in industrialized countries: in 5–10 % of individuals, as detailed elsewhere in this special issue. Isolated IDA can be detected by a complete blood count, and iron status based on the reticulocyte count or reticulocyte production index (RPI), erythrocyte indices, ferritin (FT), and transferrin saturation (TSAT). Typically, IDA is an isolated hyporegenerative microcytic hypochromic anemia, with reduced FT concentration and TSAT as indicators of a depletion of iron stores and serum iron, respectively [12–15]. The RPI can easily be estimated by one of two established formulas (Fig. 2).

ID results in difficulties regenerating epidermis and mucosal epithelia, while also affecting the clinical course of associated chronic diseases. For instance, ID has negative effects on mitochondrial respiration and tissue oxygen consumption and, thus, on cardiac function and the clinical course of congestive heart failure (CHF) [16–18]. The importance of anemia for CHF is underscored by a linear increase of mortality with declining Hb levels [19–21]. Likewise, parenteral iron substitution has been found to improve the clinical course of CHF in patients with coexisting ID [22–24].

## Anemia of inflammation

AI can be viewed as a spectrum of acute and chronic forms of anemia whose common pathophysiological denominator is their occurrence as a result of immune activation [25, 26].

Acute and chronic infections, inflammatory disorders, and malignancies are the principal disease types underlying AI. However, AI shares features with the renal anemia observed in patients with chronic kidney disease (CKD), the anemia in patients with chronic obstructive pulmonary disease (COPD), the anemia in patients with CHF without or with cardio-renal syndrome, and the anemia of the elderly [23, 27, 28].

The anemia of critical illness occurring after acute events such as major surgery, severe trauma, myocardial infarction or sepsis may be classified as a specific acute form of AI. Moreover, some features of AI also characterize the anemias occurring in hematologic disorders such as multiple myeloma or malignant lymphoma [29–32].

In addition, combined forms of IDA and AI may be present. This scenario is typically observed in inflammatory bowel disease (IBD) or gastrointestinal or urogenital malignancy. Mucosal erosions and ulcerations are associated with recurrent bleeding episodes and lead to a substantial loss of iron, since 0.5 mg of iron are contained within the Hb of 1 ml of blood. At the same time, the underlying disease provides an inflammatory stimulus for the sequestration of iron in the MPS. Moreover, menstruation, hemodialysis, the requirement for repetitive blood sampling, and anticoagulant or antiplatelet drugs may contribute to iron loss in CKD and other chronic diseases.

## Multiple players in the pathophysiology of AI

### Immune cells

The activation of immune cells by infectious agents, auto-antigens, or neoplastic cells initiates and maintains the development of AI by several mechanisms which coexist and are cross-regulatory (Fig. 1b). The excessive production of inflammatory mediators diverts iron to the MPS, rendering it relatively unavailable for erythroid progenitors [33]. A paradigm for such a mediator is hepcidin anti-microbial peptide (HAMP). HAMP is the hormonal negative-feedback regulator of serum iron, as it limits iron-fluxes to the circulation. Upon iron excess or inflammation, HAMP is produced by hepatocytes and, in much smaller quantities, by immune cells and other cell types. HAMP’s specific receptor is FPN1, whose only known function is to act as an export protein for ionic iron. Binding of HAMP to FPN1 tags the latter for internalization from the cell membrane and for lysosomal degradation [34].

Activation of pattern recognition receptors such as Toll-like receptor (TLR)-4, as well as pro- and anti-inflammatory cytokines regulate HAMP expression, while similar pathways control transcriptional expression of iron transporters transferrin receptor (TFR)-1, DMT1, and FPN1, as well as the iron storage protein FT [26].

For instance, lipopolysaccharide as a component of the Gram-negative cell wall enhances HAMP production while stimulating DMT1 expression in myeloid cells, thereby favoring iron sequestration [35]. In parallel, interleukin (IL)-10 increases TFR1 and FT transcription, which may aggravate AI in patients with IBD [36].

Increased HAMP levels are also well documented in infections, rheumatoid disorders, and IBD. Furthermore, in almost all patient cohorts, HAMP concentration positively correlates with disease activity linking the extent of inflammation to the severity of iron sequestration in the MPS [37–43].

### Liver

The liver is a key organ initiating and maintaining AI [12]. Hepatocytes are the key source of HAMP, while Kupffer cells (KC) are a major site of inflammation-driven iron storage. Interestingly, KC dampen HAMP production in homeostatic conditions but may be required for inflammation-driven HAMP secretion [44, 45]. IL-6 is essential for the up-regulation of HAMP upon inflammation and IL-6 blockade for the treatment of rheumatoid arthritis lowers both disease activity and circulating HAMP levels [46, 47]. TF is a major product of hepatocytes and one of a limited number of negative acute phase reactants. IL-6 and other pro-inflammatory cytokines result in a downregulation of TF expression in the liver, thus reducing the serum’s capacity to transport iron [48]. This mechanism may additionally contribute to iron sequestration in the MPS. Since TF-bound iron and TFR1 form the key mechanism of iron uptake for erythroid progenitors, a central role for the development of AI is implicit. TFR1 is also expressed by neoplastic cells in solid tumors and hematologic malignancies, including chronic lymphocytic leukemia, suggesting that inflammation associated with malignant diseases may also limit iron availability for cancer cells [49, 50]. However, potential functional consequences for tumor-associated monocytes/macrophages (TAM) are not sufficiently addressed. In addition, several pathogens are able to acquire TF-bound iron [51–54]. Therefore, the reduction of serum TF appears to be one of the mechanisms of microbial iron withdrawal [54–57].

Serum iron (TF-bound iron), the amount of stored iron (FT-stored iron), and the iron demand for erythropoiesis are key variables that are integrated by hepatocytes to adapt HAMP production to current metabolic needs. Serum iron levels are sensed by a machinery involving TFR1, TFR2, and the hemochromatosis-associated HFE protein [58]. However, in being the primary iron source for erythropoiesis, TF also indirectly regulates HAMP expression via erythroid progenitor-derived mediators, suggesting that the pathways of HAMP regulation are interconnected [59–61].

An increase in the erythropoietic activity as observed after blood loss or erythropoietin (EPO) administration suppresses HAMP production [62]. Part of this effect may be mediated via erythroferrone (ERFE), a lack of which delays the recovery from AI in a mouse model [63]. Growth-differentiation factor (GDF)-15, whose levels are increased in thalassemia and AI with or without ID, also inhibits HAMP expression [60, 64]. Hypoxia has a similar effect on HAMP that is mediated via platelet-derived growth factor isoform BB (PDGF-BB), which may enable the required increase of Hb levels at high altitude [65].

Iron accumulation in the liver induces bone morphogenetic protein (BMP)-6, which is essential to maintain body iron homeostasis. BMP6 binds to a heterodimeric receptor complexed with hemojuvelin (HJV) and matriptase-2 (the gene product of TMPRSS6), and stimulates HAMP expression [66, 67]. Notably, BMP6 is primarily produced by non-parenchymal liver cells and may act in a paracrine manner on adjacent hepatocytes [68].

In the context of inflammation, IL-6 and IL-22 stimulate HAMP expression via specific receptors signaling through signal transducer and activator of transcription (STAT)-3, while alpha-1 antitrypsin may do so via HJV and matriptase-2 [69–71]. However, inflammation also feeds into the BMP6 signaling pathway, adding further complexity; not only to the regulation of iron homeostasis, but also to the pathophysiology of AI and the clinical interpretation of iron indices [72].

In their reproductive years, women have an increased iron demand. Estradiol, whose levels increase after menstrual bleeding during the first half of the menstrual cycle (follicular phase) until ovulation, inhibits HAMP transcription in hepatocytes, which may allow for higher intestinal iron absorption to compensate for the average 20–80 ml of monthly menstrual blood loss [73, 74]. In contrast, progesterone, which rises after ovulation and dominates the second half of the cycle (luteal phase) until menstrual bleeding, rather stimulates HAMP expression [75]. Given the resulting fluctuations of HAMP and iron indices, the last five days of the menstrual cycle have been proposed for blood sampling to allow for a more representative evaluation of iron status in women [76].

Recently, the concept has emerged that drugs may have undesired side effects on iron homeostasis, since the mTOR inhibitor rapamycin may increase HAMP levels after heart transplantation, thus inducing a functional ID and anemia [77].

### Spleen

The spleen contributes to the pathogenesis of AI as site of iron retention in macrophages. Furthermore, splenomegaly may result in hypersplenism and a reduced half-life of red blood cells (RBC) as a consequence of the increased RBC elimination by red pulp macrophages (RPM). Similarly, evidence from mouse models suggests that increased erythrophagocytosis contributes to the rapid Hb drop in acute and subacute forms of AI [78, 79]. Under conditions of excessive inflammation as seen in sepsis patients, reactive oxygen intermediates may further accelerate RBC damage and their removal by complement-dependent mechanisms [80, 81].

### Kidney

While hepatic HAMP formation is increased during inflammation, EPO production in the kidney is subject to inhibition by inflammatory mediators such as tumor necrosis factor (TNF) and IL-1 [82–84].

CKD with a glomerular filtration rate (GFR) < 40 ml/min/m^2^ results in insufficient or deregulated production of EPO and of 1, 25-dihydroxy-cholecalciferole, both of which are negative regulators of HAMP [85, 86]. Theoretically, for the assessment of whether the renal EPO response is adequate in AI, the EPO concentration as measured should be corrected for the actual Hb level (comparable to RPI for the correction of reticulocyte counts). However, no consensus exists on a correction formula for EPO for subjects with normal renal function or for CKD patients [84, 87].

Independently, glomerulopathy may result in proteinuria and the loss of the 80-kD serum protein TF, which is the major shuttle between compartments of iron absorption (intestine)/iron recycling (MPS) and the erythron. While isolated antibodies to TF may lead to IDA, such auto-antibodies have not yet been reported in systemic autoimmune diseases. However, it is known that a functionally distinct type of anti-TF antibodies in monoclonal gammopathies may result in hyperferritinemia and increase of hepatic iron storage [88, 89].

### Erythron

A resistance of the erythron to EPO is another mechanism underlying AI, since it reduces the erythropoietic drive even in the setting of normal or adequately increased serum EPO concentrations. Part of this may be attributed to downregulation of the EPO receptor on erythroid cells by interferon (IFN)-γ [90]. Furthermore, a range of inflammatory mediators including TNF, IL-1, IFN-γ, and reactive intermediates inhibits the proliferation and differentiation of erythroid progenitors or induces their apoptosis [91–94]. These pathways ultimately culminate in an insufficient renal EPO response and hematopoietic EPO resistance further aggravating anemia in AI [95].

Numerous infectious agents (e. g., parvovirus B19 and human herpes virus-6) and neoplastic cells may infiltrate the bone marrow, which eventually disturbs erythropoiesis by several mechanisms, including direct damage to erythroid cells and putative negative effects on the microenvironment and the stem cell niche. In addition, there may be direct toxic effects of drugs including chemotherapeutics and of radiation therapy on hematopoietic stem/progenitor cells. Cytopenia, including anemia, is a concern of methotrexate treatment for rheumatoid arthritis [96]. However, immunological deregulation induced by biologics such as anti-TNF therapy may also induce aplastic anemia [97].

While in its classical form AI constitutes a hyporegenerative anemia, hemolysis may contribute to the development of AI or aggravate its degree in several settings. For instance, several bacteria including *Staphylococcus aureus *produce hemolysins [98]. These destroy RBC, liberating heme for its uptake into bacteria by specific receptors. Different mechanisms of heme iron acquisition are exploited by intraerythrocytic infectious agents such as *Plasmodium *[99]. In addition, malaria induces HAMP, suggesting that iron sequestration is a major contributing factor to malarial anemia [100, 101]. Elevated HAMP levels have also been reported in patients with HIV (Human immunodeficiency virus) infection, in which they are associated with anemia and independently predict mortality [102]. While auto-antibodies against RBC can be induced by acute Epstein–Barr virus and *Mycoplasma pneumoniae* infections resulting in cold agglutinin disease, auto-immune hemolysis may also occur in the setting of chronic infections or as a side effect of medication [103]. In addition, the life span of circulating RBC may be negatively affected by inflammatory mediators such as TNF and by mechanical stress [104]. Therefore, hemolysis may also contribute to AI in conditions such as CHF associated with mechanical valve replacement or endocarditis, or when microangiopathy is present. However, due to fluid retention, the degree of anemia tends to be overestimated in CHF patients.

### Others

Similar to the concurrent presence of absolute ID in the setting of AI, deficiencies in other nutrients essential to erythropoiesis, such as folate and vitamin B12, may be contributory. For instance, celiac disease may cause profound malassimilation of various nutrients or poor food intake may aggravate the anemia of the elderly. Particularly in elderly patients, anemia due to clonal hematopoietic diseases, including myelodysplastic syndromes (MDS), has to be considered as well.

## Current and promising diagnostic tools

### Complete blood count, reticulocyte production index, and red blood cell indices

Both IDA and AI typically manifest as isolated anemia. As detailed elsewhere in this special issue, both absolute ID and inflammation can also result in thrombocytosis due to the effects of altered thrombopoietin, EPO, and IL-6 levels on megakaryopoiesis [105–107]. In addition, the disorders underlying AI or the immune-modulatory therapy required for their control can affect circulating leukocyte numbers [108]. A differential blood count can be recommended for unclear cases of anemia where monoclonality may be suspected as an underlying disease (Fig. 2). Serum protein electrophoresis and bone marrow aspiration or trephine biopsy may reveal additional diagnostic clues. The reticulocyte count allows for the differentiation of hyporegenerative anemias (disorders of erythroid proliferation and maturation) vs. regenerative anemias (hemolysis or hemorrhage). However, to account for the increased proportion of reticulocytes in anemia and the increased presence of prematurely released reticulocytes in the circulation, the RPI should be calculated (Fig. 2).

Erythrocyte staining indices do not define the cause of anemia, but they may be helpful during the diagnostic workup. IDA is a microcytic hypochromic anemia, while AI may be microcytic hypochromic or normocytic normochromic in appearance. High normal to elevated MCV and MCH may be due to a complex metabolic disorder (e. g., in alcoholism), severe nutrient deficiency (e. g., in celiac disease), or an alternative diagnosis such as MDS.

In addition, clinical signs along with the measurement of TSH (Thyroid stimulating hormone) and PTH (Parathyroid hormone) will help to rule out endocrine disorders (specifically hyperthyroidism, hypothyroidism, panhypopituitarism, and hyperparathyroidism) as the cause of a hyporegenerative, normocytic normochromic anemia.

### Ferritin and transferrin saturation

In IDA, serum FT and TFS may enable an accurate interpretation of body iron status. A reduction in serum FT below 30 ng/ml shows ID with high diagnostic accuracy because a strong correlation exists between serum FT and the body’s total iron storage. It is generally assumed that for each 1 ng/ml of serum FT, 10 mg of iron are stored in tissues and organs. Serum FT appears to be iron-poor and mainly derived from macrophages [109].

Serum FT levels in the setting of inflammation are more difficult to interpret as a range of stimuli result in altered production of FT. Therefore, the clinical presentation, along with markers of inflammation such as C‑reactive protein or IL-6, needs to be taken into account. The appearance of hyperferritinemia >200 ng/ml in the context of a decreased TSAT is suggestive of immune-driven iron sequestration. This may be indicative of inflammation, cancer, infection, or liver disease. Extraordinarily high FT levels have been documented in patients with adult-onset Still’s disease or hemophagocytic syndrome [110, 111].

In an attempt to transport the available iron as efficiently as possible, serum TF is increased in ID, resulting in a TSAT < 16 %. Similar levels are observed in AI because TF is a negative acute-phase reactant (see above).

Hyperferritinemia in the context of an increased TSAT of >45 % should prompt evaluation for primary or secondary iron overload. In the context of microcytic anemia and Mediterranean or Asian descent, thalassemia is a valid differential diagnosis. In the absence of anemia, HFE-associated hemochromatosis or dysmetabolic iron overload are possible explanations for pathologically increased FT and TSAT.

### Soluble transferrin receptor and ferritin index

TFR1 is the key receptor for iron acquisition by erythroid cells. Its soluble form (sTFR) can be measured in the serum and it reflects ID and erythropoietic activity. sTFR is increased in ID, hemolytic anemias, thalassemia, and some hematologic malignancies, while its levels tend to be normal in AI [112]. Therefore, an increased sTFR in the setting of AI suggests the presence of additional absolute ID. However, the use of sTFR is limited by the lack of its standardization and the fact that age, ethnicity, and inflammation influence its normal range [113].

The ferritin index (FTI) may also be helpful in the differential diagnosis of AI and combined IDA/AI. However, the lack of standardized tests for sTFR prevents its broad recommendation. The FTI is calculated from the sTFR divided by the logarithm of serum FT (Fig. 2). In patients with chronic diseases and AI, an increased FTI suggests the concurrent presence of absolute ID requiring correction. However, the cutoff value is largely dependent on the specific diagnostic test used [114–116]. Therefore, at the current stage of research we are unfortunately not able to provide a universally applicable algorithm for the differentiation between isolated AI and anemia with combined functional and absolute ID.

### Content of reticulocyte hemoglobin, percent of hypochromic erythrocytes, and zinc protpoporphyrin

Content of reticulocyte hemoglobin (CHr), percent of hypochromic erythrocytes (%HYPO), and zinc protoporphyrin (ZnPP) allow for the prediction of iron availability for erythropoiesis, but have little to no role in the differentiation between IDA and AI.

The content of Hb in reticulocytes correlates with the availability of iron for erythropoiesis. A CHr < 26 pg suggests iron-limited erythropoiesis, as observed in both IDA and AI. In response to iron substitution, it is one of the first parameters to respond with an increase. A lack of this predicted response raises the concern of an alternative diagnosis, unless CKD is present and EPO deficiency awaits correction.

HYPO is defined as the relative number of hypochromic RBC with a Hb content <28 pg. A HYPO >10 % indicates iron-deficient erythropoiesis due to IDA or AI.

As erythropoiesis becomes iron-deficient, the erythroid enzyme ferrochelatase incorporates zinc instead of iron into protoporphyrin-IX. Since ZnPP and heme are analogues, an increase in the ratio of ZnPP/heme indicates ID for erythropoiesis and is observed in IDA, AI, MDS, and sideroblastic anemias, including the form secondary to lead intoxication [117]. This highlights the lack of specificity of this set of parameters for the differential diagnosis of anemias.

### Hepcidin and its regulators

Hepcidin (HAMP) may be helpful in the differential diagnosis of anemias, as well as in the assessment of therapeutic options. For instance, HAMP is suppressed in IDA, in the normal range in IDA/AI, and elevated in AI [112, 118]. High HAMP at the time of initiation of therapy with ESA may predict poor treatment response in AI [119]. In addition, high HAMP predicts poor response to oral iron in IDA patients [120]. In CKD patients, the predictive power of HAMP for the indication for iron therapy is limited [43, 121, 122]. Attempts have been undertaken to harmonize the different diagnostic methods for hepcidin determination to allow the broad clinical use of this method [123]. GDF15 is normal in IDA and elevated in AI and IDA/AI [64].

In the future, information technology may provide us with software based on complex algorithms for a more accurate assessment of iron status and, just as importantly, guide therapy for the most appropriate treatment. For instance, we may witness that mobile applications based on a combined panel of HAMP, EPO, ERFE, GDF15, BMP6, and other parameters, such as high sensitive CRP and IL-6, enter clinical routine.

## Treatment options

Since the AI is a direct consequence of an active immune-driven disease, its first-line therapy is treatment of the underlying condition. However, the subsequent therapeutic approach to AI remains a matter of debate and ongoing clinical trials. Iron supplementation, ESA, and transfusion of packed RBC are the current specific treatment options for AI.

It is generally assumed, similar to the hypoferremia of the acute phase response, that AI is the pathophysiological consequence of the body’s attempt to reduce the availability of iron for infectious agents. Therefore, there is the concern that iron supplementation may stimulate pathogen proliferation or result in a flare of an underlying inflammatory disorder or malignancy [54, 124, 125]. Similarly, ESA and RBC transfusions may have adverse immune-modulatory effects [126–129]. The target therapeutic Hb levels have not yet been defined in prospective trials; however, data from studies in patients with anemia and CKD or cancer suggest a slightly anemic target range between 11–12 g/dl to be safe [130, 131].

### Iron preparations

Isolated IDA can often be prevented by iron fortification/supplementation and, once it has manifested, is preferentially treated by oral iron salts. For instance, approximately 100 mg of elemental iron contained in 300–350-mg ferrous sulfate preparations can be prescribed as a daily dose. These iron salts are absorbed by the sequential action of DMT1 and FPN1 and associated oxidoreductases, but have a low bioavailability. However, products using heme rather than ionic iron have entered the market. These are absorbed by alternative pathways that are incompletely characterized, since the proposed solute carrier SLC46A1 absorbs folate more efficiently than heme [132, 133]. A recent study in non-anemic young women with ID has shown that a single morning dose of 40–80 mg ferrous sulfate resulted in adequate iron absorption yet elicited a transient rise in serum HAMP levels, which argues against twice-daily dosage. Whether or not alternate day supplementation provides a benefit awaits investigation in prospective trials [134].

Parenteral iron supplementation is an alternative to consider, especially when a rapid correction is needed, or gastrointestinal (GI) malassimilation or active inflammatory disease dampens dietary iron absorption in AI [135, 136]. Also, in patients with intolerance to oral iron supplements, parenteral iron is the therapy of choice. Currently, six different forms of parenteral iron are available for clinical use, i. e., ferric carboxymaltose, ferumoxytol, iron dextran, iron gluconate, iron isomaltoside, and iron sucrose. These represent macromolecules in which iron is complexed to saccharides. The complexes are endocytotically taken up by the MPS and ionic iron is distributed into the circulation via FPN1 by macrophages [137]. Concerns have been raised regarding the risk of severe anaphylactic reactions when using intravenous iron preparations. However, these are infrequent and specific precautions are recommended in at-risk patients, to minimize the occurrence of such adverse events [138]. In addition, parenteral iron supplements harbor an intrinsic risk of inducing hypophosphatemia. Therefore, serum phosphate may be measured when erythropoietic and iron indices are determined to evaluate the response to treatment [139].

Despite the fact that the MPS has a lower threshold to respond to increased HAMP than have duodenal enterocytes, parenteral iron remains effective when intestinal iron absorption is hampered by immune activation [140]. Since parenteral iron stimulates HAMP secretion, as documented in hemodialysis patients, frequent administration of low doses may be beneficial [141]. Prospective trials are required to optimize treatment regiments to ensure adequate efficiency of parenteral supplementation in different clinical settings.

In the context of AI, parameters which predict the subsequent response to EPO therapy are being evaluated in prospective studies. In CKD patients, iron therapy is specifically recommended to replenish stores prior to initiation of ESA therapy. TSAT < 20 % and FT < 100 ng/ml have been proposed as cutoffs for absolute ID in non-dialysis CKD patients [142]. Further details are reviewed elsewhere [130].

### ESA

Currently, the arm of therapy with ESA remains limited to EPO analogues, as the synthetic EPO receptor agonist peginesatide has been taken off the market because of rare anaphylactic reactions [143].

Many subjects with AI who are under causative treatment for their underlying condition do not have an adequate Hb response to iron therapy. EPO resistance of the erythron or renal EPO deficiency may be present, such that ESA should be considered as add-on therapy for anemia. Specific studies have been conducted in patients with AI in the setting of rheumatoid arthritis or HIV infection, in which EPO levels <500 U/L predicted a response to ESA administration [144, 145]. Standard starting doses of EPO are 100–150 U/kg, administered subcutaneously three times a week, although higher doses may be required for individual patients. As Hb levels increase during efficient EPO therapy, iron parameters should be monitored and iron supplemented in order to maintain a TSAT ≥20 % and a FT ≥100 ng/ml for a sufficient Hb response. In dialysis patients, higher FT target levels have been suggested [146]. In MDS, ESA are in wide clinical use despite of the fact that official approval of this approach is still pending [147]. The specific regimes and pitfalls in ESA therapy are reviewed elsewhere [130, 131, 148, 149].

### The HAMP–FPN1 axis as target

Given its important role for iron-sequestration in AI, HAMP and its receptor FPN1 are attractive targets for therapeutic interventions. Different pharmaceutical preparations including antibodies, antichalins, Spiegelmers, thiamine derivatives, and heparin derivatives can bind and neutralize HAMP [150, 151]. Moreover, direct blockage of HAMP expression via sHJV or BMP signaling inhibitors have shown efficiency in blocking HAMP function and ameliorating anemia [152]. Some of these treatments are already being evaluated in clinical trials [153–158]. Moreover, an FPN1 stabilizing antibody is currently also under investigation [159].

Since the HIF–EPO axis forms an alternative targetable pathway, prolyl hydroxylase inhibitors have entered clinical trials. This class of drugs can be orally administered and protects HIF from degradation, which increases EPO levels and erythropoietic iron availability [160].

## Summary

The precise differential diagnosis between IDA, AI, and a combination of both forms is of clinical importance because of differing treatment strategies. Currently, the lack of data from prospective clinical trials prevents definitive recommendations on diagnostic algorithms and prognostic indices. These problems are aggravated by the lack of standardization in otherwise promising tests, such as measurement of sTFR. However, within the next few years, standardized tests for novel parameters such as HAMP or ERFE will become available for more accurate differential diagnosis, stratification of treatment indications, and prediction of therapeutic response. Furthermore, the HAMP–FPN1 axis continues to receive a lot of attention as a therapeutic target. Blocking HAMP expression or processing, neutralization of circulating HAMP, and blockage of its interaction with FPN1 are under active investigation for the treatment of AI.

Moreover, different forms of AI may have to be taken into account. Dependent on the underlying conditions and dominant pathophysiological pathways, a more personalized approach to optimal management of distinct forms of anemias will be required.
